# Trends in GP prescribing of psychotropic medications among young patients aged 16–24 years: a case study analysis

**DOI:** 10.1186/s12888-017-1375-2

**Published:** 2017-06-06

**Authors:** Bianca Brijnath, Ting Xia, Lyle Turner, Danielle Mazza

**Affiliations:** 1 School of Occupational Therapy and Social Work, Faculty of Health Sciences, Curtin University, Building 401, Bentley Campus, Perth, 6152 Australia; 2 Department of General Practice, School of Primary Care, Faculty of Medicine Nursing and Health Sciences, Monash University, Melbourne, Australia

**Keywords:** Australia, General practitioners (GPs), Youth, Mental health, Psychotropic, Prescribing

## Abstract

**Background:**

Current clinical guidelines recommend non-pharmacological interventions as first-line treatments for young patients aged 16–24 years with a mental health condition (MHC). However, several studies have noted increasing trends in psychotropic prescribing for this age group, especially in antidepressant prescribing. In Australia, the vast majority of psychotropic medications prescribed to young people come from the general practice setting. To assess whether Australian General Practitioners (GPs) are prescribing in accordance with clinical guideline recommendations, this study examined trends in GP prescribing of psychotropic medications to young patients aged 16–24 years.

**Methods:**

We performed a retrospective analysis of routine general practice data from 9112 patients aged 16–24 years with a MHC. Data were extracted from the Melbourne East Monash General Practice Database from 1/01/2009 to 31/12/2014. The main outcome measures included the number of consultations for patients with MHCs, psychotropic prescribing by GPs, and patient characteristics associated with the likelihood of being prescribed a psychotropic.

**Results:**

In total, 9112 out of a total of 77,466 young patients were identified as having a MHC in this study, and 11,934 psychotropic prescriptions were provided to 3967 (43.5%) of them over the study period. Antidepressants accounted for 81.4% of total psychotropic prescriptions, followed by anxiolytics (9.6%) and antipsychotics (9.0%). The number of prescriptions issued to individuals with MHCs increased over time. Women and patients aged 21–24 years had higher incidence rates for prescription than men and those aged 16–17 (IRR: 1.15, 95% CI 1.08–1.22, IRR: 1.93, 95% CI 1.750–2.11).

**Conclusions:**

Our findings demonstrate an increasing trend in GP prescribing of psychotropics to young people over the study period with higher levels of prescribing to women and those 21–24 years of age. Although GP prescribing corresponded with guideline recommendations on the whole, there were discrepancies between GP’s antidepressant prescribing and guideline recommendations, reasons for which were unclear. Research is needed to investigate GPs decision-making processes underlying their prescribing, to target interventions to improve existing data in GP records to improve management, and to identify areas of further training if needed to facilitate greater concordance between clinical practice and guideline recommendations.

## Background

In Australia, over 89% of antidepressants and 70% of antipsychotics prescribed to young people aged 15 to 24 are prescribed by General Practitioners (GPs) [1]. UK, Canadian, and Australian studies show that GPs face many challenges in managing mental illness for this patient group, such as grappling with the unique behavioural and biological changes associated with adolescence; navigating the difficulties associated with triadic consults (i.e., where a third party such as a parent or friend might be present during the GP consult with a young person); negotiating different expectations by young people and families around socially accepted behaviours regarding alcohol and substance use, use of digital media, and relationships between teenagers and parents; working out ways to forge effective and trusting therapeutic relationships with young people; [2] and treating severe and persistent mental illness [3, 4]. Studies show that GPs are apprehensive about over-medicalising young patients [5] and are less likely to prescribe psychotropics if their young patient’s hold negative views about psychotropics [6], express a preference for non-pharmacological treatments [7], or are new patients at their clinic [8]. Health warnings also reduce the likelihood of prescriptions; for example, the imposition of US and European regulator suicidality warnings on Selective Serotonin Reuptake Inhibitors (SSRIs) use in young people resulted in a decline in SSRI prescriptions among US and Dutch clinicians [9].

Current guidelines for youth mental health in Australia recommend prescribing only those psychotropics with the best evidence base and safest profile in the lowest effective dose for the shortest time possible (Table 1) [10–12]. These guidelines concord with guideline recommendations from the UK and US, where the philosophy “start low and go slow” underlies nearly all recommendations regarding psychotropic prescribing in young people [13, 14]. Previous analysis from two Australian national general practice clinical audits (1998–1999 and 2000–2002) showed GP psychotropic prescribing was conservative for youth mental health with non-pharmacological treatments alone, or in combination with psychotropics, being the preferred option [15]. However, this data is over 15 years old and changes in Australian primary care, such as the implementation of the Better Access to Mental Health Care Scheme (in which GPs can refer patients to mental health specialists for up to 10 free sessions per year), and availability of new psychotropics (e.g. Serotonin-norepinephrine reuptake inhibitors, or SNRIs) may have influenced GP prescribing patterns for youth mental health. Longitudinal trends in GP prescribing [16] and pharmacy dispensing [17] for psychotropic medications suggest this is the case for the general adult population. However, less is known about GP psychotropic prescribing to young people. Accordingly, in this paper, we conducted a non-comparative case study analysis to examine trends in GP psychotropic prescribing to patients aged 16–24 years over a 5 year period.

**Table 1:** Summary of guideline recommendations for psychotropic prescribing for youth mental health [10–12]

• Non-pharmacological interventions are the first line of treatments for YMH	
• Medication treatment should follow thorough assessment and diagnosis and be part of a comprehensive care plan	
• Only those psychotropics with the best evidence base and safest profile should be prescribed	
• Prescriptions should be in the lowest effective dose for the shortest time possible	
• Fluoxetine, the only SSRI with a strong evidence base, should be considered following unsuccessful psychological therapy for reduction of moderate to severe depressive symptoms in adolescents	
• Benzodiazepines are generally not recommended for use in children.	
• Ongoing monitoring following psychotropic prescriptions is critical.	

## Methods

Data were drawn from the Melbourne East Monash General Practice Database (MAGNET) a collaboration between Monash University and the Melbourne East GP network. MAGNET comprises data routinely collected from the computerised medical records of patients attending 50 general practices within the inner eastern Melbourne region and is ethics approved from Monash University.

We analysed data from 1 January 2009 to 31 December 2014 for all patients aged 16–24 years at the time of consultation. Eligibility criteria for inclusion in the study were: Patients had a diagnosis of a mental health condition (MHC) in their clinical record and/or had been prescribed a psychotropic medication during the study period. Patients were identified as having a mental health diagnosis through examination of coded diagnoses attached to their computerised medical record [18].

Diagnoses were coded into high-level categories reflecting the primary problem description or diagnosis using a computerised algorithm [18]. Psychotropic medications were coded according to the Anatomical Therapeutic Chemical classification system. This system is used for the classification of active ingredients of drugs according to the organ or system on which they act and their therapeutic effect. Medication records were excluded for patients aged above 25 at the time of prescription.

For each patient we extracted demographic characteristics (age, gender, socioeconomic status, postcode, smoking, and alcohol status), consultation information (the clinic location and the consultation dates), and information about prescribed medications (the prescription data and the classification of active ingredients of medications). As referral data were incompletely recorded, they were not extracted for this study. Patients’ residential postcode was used to identify the corresponding Index of Relative Socioeconomic Disadvantage, a proxy for the socioeconomic status of each patient [19].

Descriptive analysis was used to describe the annual trends in the number of consultations, patients with MHCs, number of psychotropics prescribed by GPs, and annual trend in the number of psychotropic prescriptions across sub-categories – i.e. antidepressants, anxiolytics, antipsychotics, and antiepileptics. Negative binomial regression was used to explore changes of prescription patterns (annual psychotropic prescriptions per head of people with a MHC from GP clinics involved in MAGNET) over the study period, and to determine what patient characteristics were associated with the likelihood of receiving a prescription. The 0.05 level of statistical significance was adopted for each test. Results for the regression models were expressed as incidence rate ratios (IRR) with 95% confidence interval (CIs). All analyses were conducted using Stata v13.0.

## Results

From 2009 to 2014 there were 605,417 GP consultations for 77,466 patients aged 16–24 years with an average of 7.8 consultations per patient (95% CI = 7.73–7.91). Of 77,466 patients, 7892 (10.1%) had been diagnosed with a MHC (63.9% of these were female). Additionally, 1220 patients had been prescribed a psychotropic without a MHC diagnosis (60.9% of these were female). Thus a total of 9112 (11.8%) young patients were identified as potentially having a MHC in this study.

Among those 9112 young patients with MHC, consultations with female patients (122,645) were higher than for male patients (50,241). Female patients averaged 21.1 consultations (95% CI = 20.4–21.7) during the period 2009–2014, compared to 14.8 consultations for male patients (95% CI = 14.2–15.4). The majority of young patients who had at least two consultation records (78.9%) visited only one medical clinic for their consultations. Table 2 presents these patient’s demographic characteristics and mental health status. Nearly half of MHC patients (45.8%) were non-smokers, 16.8% and 16.0% were ex-smokers and current smokers respectively (smoking status was not recorded for the remaining one fifth). Alcohol status was also not recorded for nearly 85% of the sample population; accordingly, alcohol and smoking status were not included in the analysis. Only 2.9% of patients were from areas with the lowest Index of Relative Socio-Economic Disadvantage quintile and about 88% were from the top two quintiles.

**Table 2:** Patient’s demographic and mental health status

	Total (*N* = 9112)	
	N	%
Gender (10missing)		
Female	5774	63.7
Male	3328	36.2
Smoking status		
Non-smoker	4173	45.8
Ex-smoker	1531	16.8
Current smoker	1456	16.0
Not recorded	1952	21.4
Alcohol status		
Non-drinker	358	3.9
Drinker	1054	11.6
Not recorded	7700	84.5
Index of disadvantage quintile^a^(59 mssing)		
1	261	2.9
2	298	3.3
3	560	6.2
4	2580	29.0
5	5311	58.7
Mental health status		
Diagnosed and prescribed psychotropic	2747	30.1
Diagnosed and not prescribed psychotropic	5145	56.5
Not diagnosed and prescribed psychotropic	1220	13.4
Mental health-related prescriptions	11,934	

^a^refer to Socio-Economic Indexes for Areas by postcode

**Fig. 1:**
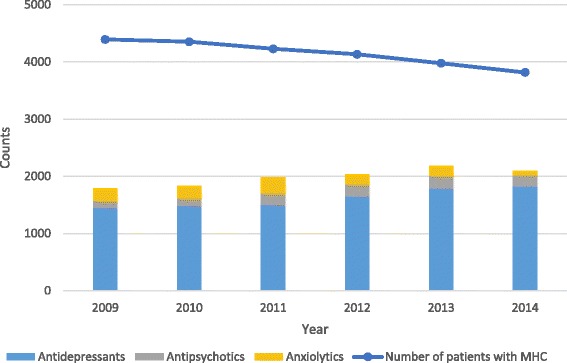
Number of visiting patients with MHC and psychotropic prescriptions (by medication class) in inner eastern Melbourne, 2009–2014

Over the study period 11,934 psychotropic prescriptions were initially provided to patients having a MHC; 95% of these medication costs were subsidised (based on 2013–2014 prescription data). Among those 9112 patients with a MHC, 2747 patients (30.1%) had a diagnostic label in the record and had been prescribed psychotropics, 5145 patients (56.5%) had a diagnostic label in the record but had not been prescribed any psychotropic medication, and 13.4% of them had no diagnostic label in the record but had still been prescribed psychotropic medications (Table 2). During the study period, the number of consultations by patients with MHCs remained stable for both genders. As shown in Fig. 1, the number of patients with a MHC declined from 4394 in 2009 to 3816 in 2014. However, from 2009 to 2013 the number of psychotropics prescribed by GPs increased from 1791 to 2182, before decreasing again to 2100 in 2014. Compared to the reference year 2009, the number of prescriptions per patient slightly increased each year (IRRs 1.04, 95% CI: 0.93–1.15 in 2010 to 1.35, 95% CI: 1.21–1.50 in 2014) (Table 3). Women had a slightly higher rate of receipt of a prescription than men. Patients aged 18–20 and 21–24 were 1.58 and 1.93 as likely respectively to be prescribed psychotropics when compared to those aged 16–17 (Table 3).

**Table 3:** Amount, prescription rate per head of people with mental health condition, IRR of number of prescriptions in inner eastern Melbourne region, 2009–2014

Category	Number of prescriptions	Number of patients	IRR	95% CI	*p*-value
Year of visit					
2009	1791	4394	1 (ref)		
2010	1837	4353	1.04	0.93–1.15	0.490
2011	1987	4230	1.15	1.04–1.27	0.009*
2012	2037	4133	1.21	1.09–1.34	< 0.001*
2013	2182	3977	1.35	1.21–1.49	< 0.001*
2014	2100	3816	1.35	1.21–1.50	< 0.001*
^a^Gender					
Male	3850	3328	1(ref)		
Female	8078	5774	1.15	1.08–1.22	< 0.001*
Age group					
16–17 years	1074	2455	1 (ref)		
18–20 years	3442	4093	1.58	1.43–1.75	< 0.001*
21–24 years	7418	6617	1.93	1.75–2.11	< 0.001*

^a^10 records with missing data were removed from analysis

* *p* < 0.05 level

Antidepressants were the most commonly prescribed psychotropic (81.4%), followed by anxiolytics (9.6%) and antipsychotics (9.0%) (Table 4). Antidepressant prescriptions increased by nearly 30% across all age groups from 2009 to 2014 (Fig. 1), contributing to the increase in overall prescriptions across the study period. Conversely, anxiolytics, which constituted 11.6% of total prescriptions in 2009 became the least commonly prescribed psychotropic in 2014, only constituting 3.6% of the total (Fig. 1).

**Table 4:** Psychotropic prescriptions, by Anatomical Therapeutic Chemical (ATC) classification of medication prescribed in in inner eastern Melbourne region, 2009–2014

Medication level 3	Medication level 4	N	% Subtotal	% Total
Antidepressants	Selective Serotonin Reuptake Inhibitors (SSRIs)	7288	75.1	81.4
	Serotonin-norepinephrine reuptake inhibitors (SNRIs)	1169	12.0	
	Other Antidepressants	643	6.6	
	Tricyclic antidepressant.	583	6	
	Monoamine Oxidase Inhibitors	26	0.3	
Subtotal		9709		
Anxiolytics	Benzodiazepine Derivatives	1147	100	9.0
Subtotal		1147		
Antipsychotics	Diazepines, Oxazepines, Thiazepines and Oxepines.	922	80.3	9.6
	Lithium	54		
	Other Antipsychotics	46	4.7	
	Benzamides	29	2.5	
	Indole Derivatives	12	1.0	
	Thioxanthene Derivatives	7	0.6	
	Phenothiazines With Aliphatic Side-Chain	5	0.4	
	Phenothiazines With Piperazine Structure	3	0.2	
Subtotal		1078		
Total		11,934		

SSRIs composed 75.1% of antidepressants prescribed and included escitalopram (37.2%), sertraline (33.4%), fluoxetine (17.0%), citalopram (9.0%) and fluvoxamine (3.3%). Over 70% of the three most common SSRIs were prescribed to female patients and about 90% of them were prescribed to patients aged 18–24 years old at the visit. The most commonly prescribed SSRI to patients 16–17 years were sertraline (36.4%), fluoxetine (30.1%) and escitalopram (27.1%).

SNRIs composed 12% of antidepressants prescriptions with venlafaxine being the only SNRI prescribed. About 72% of venlafaxine prescriptions were given to females, and 91% to patients aged 16–17 years. Monoamine oxidase inhibitors were the least commonly prescribed antidepressants (Table 4). Benzodiazepine derivatives were the only anxiolytics prescribed by GPs. Diazepines and oxazepines (e.g. olanzapine and quetiapine) were the most common antipsychotics prescriptions prescribed and phenothiazine with aliphatic side-chain and phenothiazine with piperazine structure were the least common antipsychotics prescribed to young patients.

## Discussion

Our study provides insights into recent trends in GP psychotropic prescribing for to young people aged 16–24 in the inner eastern region in Melbourne, Australia.

Our study suggests that approximately 12% of the 77,466 patients in the study catchment area had been identified by their GPs as having a mental health condition. This rate is slightly higher than national data, which reported that 9% of young people aged 16–24 years had high or very high levels of psychological distress and 25% of them experienced at least one mental disorder [20]. However, as the MAGNET catchment area is socio-economically relatively prosperous, this difference may be on account of the overall higher rate of GP encounters with young people in areas of high socio-economic status compared to areas of lower socio-economic status rather than a true difference in prevalence rates [20]. Continuity of care was also high in this catchment area with this patient cohort. About 80% of patients visited only a single practice within the MAGNET group of practices in the inner eastern region of metropolitan Melbourne over the study period; overseas data indicates such relational continuity positively influences recovery from mental illness [21, 22].

Supporting previous evidence [23, 24] more females were diagnosed with a mental health condition than males, and females had greater numbers of consultations than males. This gender discrepancy could be because of a true difference in illness prevalence but it could also be an artifactual difference as female patients, compared to male patients, tend to report more mental illness symptoms (especially for depression) and present more often in general practice [1, 25, 26]. Thus, female patients’ opportunities for diagnosis and prescription are increased [1, 25, 26]. GPs, in turn, may also be over-responding to young female patient’s, and under-responding to young male patient’s distress [26]. The evidence is equivocal in this area and qualitative analyses are needed to better understand the gendered dimensions of mental illness diagnosis and psychotropic prescribing for young patients in general practice.

Patient’s age also influenced GP prescribing with those aged 21–24 almost twice as likely to be prescribed a psychotropic as those aged under 16–17. This finding is consistent with previous research suggesting that GPs are reluctant to ‘over-medicalise’ young people [5]. Low rates of prescribing to this age group may also be influenced by the negative views held by young people about pharmaceutical treatments [6] and a preference for non-pharmaceutical intervention [7]. It may also relate to fears of inducing suicidality and aggression in young people prescribed SSRIs and SNRIs with recent evidence showing a doubling of these behaviours in young people using these forms of medication [27].

In conjunction, current guidelines [10–12] recommend non-pharmacological interventions as first-line treatments. Reflecting this, our data indicated that over half of all diagnosed patients were not prescribed any psychotropic medication at all. Also concordant with clinical guidelines [10] was a marked reduction in prescription of anxiolytics or benzodiazepines over the study period, likely reflecting increased GP awareness of the potential harms of these medications and risk of abuse especially in young patients.

It is also possible that the Better Access scheme, which provides Government subsidised access to up to 10 individual visits to specialist mental health services per calendar year, may have influenced low rates of GP prescribing. However, qualitative research with GPs is needed to verify whether this is true or not. Other Australian studies [16, 17] examining adult populations show that psychotropic prescribing increased following the introduction of the Better Access scheme, especially for antidepressants. Likewise, in our study, among those receiving GP prescriptions, the psychotropic prescribing rate increased by 32% over six years across all age groups, mainly for antidepressants. Australian [28], UK [29], and US [30] studies note increased reliance on antidepressants for treating mental disorders despite concerns of low therapeutic benefit for mild-moderate depression and the potential for adverse reactions such as increased suicidality in young people [27, 31, 32]. SSRI prescriptions increased the most within the antidepressant category, specifically escitalopram, sertraline, and fluoxetine. Current clinical guidelines [11, 14] only recommend use of fluoxetine in adolescents because neither escitalopram nor sertraline have garnered sufficient evidence on which to base recommendations. In our study the rate of fluoxetine prescriptions to patients aged 16–17 years was higher (30.1%) than reported in Karanges et al.’s [1] analyses of 2009–2012 prescribing trends (19.4%), which could suggest that GP prescribing may be aligning with guideline recommendations over time. On the other hand, the overall rate of fluoxetine prescribing in our study was only 17%, superseded by escitalopram and sertraline. Reasons for the high prescription rates of these two medications in patients under 18 years is unclear; also unclear is why venlafaxine was the only SNRI to be prescribed to young patients. While venlafaxine has the highest dispensation rate of all SNRIs and is the third most commonly dispensed antidepressant in Australia, it, like other SNRIs, has been reported to cause more complex side-effects than SSRIs [17].

Our study has limitations. As mentioned, the data from MAGNET is restricted to inner eastern Melbourne region, an economically prosperous area where most patients have a high socio-economic status. This regional specificity restricts our capacity to comment on associations between low socio-economic status, MHCs, and GP prescribing. Moreover, patients may visit other practices outside the region during the study period, which limits the generalisability of the findings. Referral data were also incompletely recorded, limiting our ability to explore non-pharmacological interventions GPs utilised in their management of youth mental health. Despite a strong association between substance use and mental illness especially among young people [33], GP records were sparse with regards to their young patient’s smoking and alcohol use; such an absence could indicate that GPs fail to ask about these risk factors or that patients are reluctant to disclose their use or other mitigating factors. In any case, poor GP data records limit our ability to make inferences around mental illness, psychotropic prescriptions, and substance use. Our analysis also did not take into account repeat prescription since detailed information was not provided. Patients having MHCs were identified by coding relevant key words from GPs’ ‘free text’ manual entries in the diagnosis category of the patient’s record, and thus the quality of coding and recording were unknown. This might explain why 1220 patients were prescribed psychotropics but not diagnosed with a MHC. Coding ambiguities around diagnostic categories also limited our ability to investigate how prescribed medications directly related to diagnosed MHCs – i.e. appropriate prescribing. Another limitation should be noted is that GP might prescribe psychotropics for other health conditions (off-label prescribing), which also affect our assumption of identifying MHC patients.

## Conclusion

The degree of accord between our findings and the published literature underwrites the validity and reliability of our findings, and also provides a timely update in the scholarship around GP psychotropic prescribing for youth mental health. Our findings show that overall, GP prescribing of psychotropics for young people reflected guideline recommendations. However, there were some discrepancies between GP’s antidepressant prescribing and guideline recommendations, reasons for which were unclear. Research is needed to investigate GPs decision-making processes underlying their prescribing, to target interventions to improve existing data in GP records to improve management, and to identify areas of further training if needed to facilitate greater concordance between clinical practice and guideline recommendations. Such training might include not only more appropriate prescribing for particular age groups but also training on how to initiate conversations around de-prescribing and safely discontinuing medicines. Arguably, the latter intervention is of greater importance given the growing global reliance on antidepressants for treating common mental disorders despite limited therapeutic benefit for mild-moderate depression and its iatrogenic effects in young people [31, 32]. While the evidence is limited on how precisely to implement antidepressant discontinuation [34], unequivocally GPs have a strong role to play in the process as facilitators of safe discontinuation and managing discontinuation symptoms [35, 36]. Given that GPs are one of the most frequently utilised health care providers amongst young people [20, 37] and that the vast majority of young people are not in receipt of specialist mental health care, visits to their GP represents an important opportunity for intervention. Accordingly, GPs must be supported to continuously improve their patient care.

## Abbreviations

CI: Confidence interval
GP: General practitioner
IRR: Incidence rate ratios
MAGNET: Melbourne East Monash general practice dataset
MHC: Mental health condition
SNRI: Serotonin-norepinephrine reuptake inhibitors
SSRI: Selective serotonin reuptake inhibitors
